# Relationship Between Ocular Motility and Motor Skills

**DOI:** 10.16910/jemr.17.4.2

**Published:** 2024-10-10

**Authors:** Carmen López-de-la-Fuente, Eider Bereau Iridoy, Paula Pardo Sofín, Jose Luis Cebrián Lafuente, Víctor Berdejo, Cristina Ruiz-Garros, María José López-de-la-Fuente

**Affiliations:** Department of Applied Physics University of Zaragoza, Spain; iHealthy Research Group, IIS Aragon, University of Zaragoza, Spain; Department of Ophthalmology, Hospital University La Paz, Madrid, Spain; Department of Physiatry and Nursing University of Zaragoza, Spain

**Keywords:** Eye movement, eye tracking, saccades, Developmental Eye Movement (DEM) test, Northeastern State University College of Optometry's Oculomotor test (NSUCO), smooth pursuit, motor skills, balance, manual dexterity, ball skills

## Abstract

The primary aim of this descriptive cross-sectional study was to examine the relationship between ocular motility and motor skills in school-age children. Participants included 142 schoolchildren (mean age: 7.08 ± 0.61 years) who completed a computerised version of the Developmental Eye Movement (DEM) test while their eye movements were recorded, and Northeastern State University College of Optometry's Oculomotor test (NSUCO). Children were classified into three groups based on their level of motor performance, which was measured by the Movement Assessment Battery for Children-2 (MABC-2). The group with typical motor performance had higher percentiles for both vertical and horizontal time, fewer errors, number of saccades, fixations, and regressions, and faster test performance. Visual test results correlate with the motor assessment outcomes; correlations are weak or moderate. Our findings emphasise the interconnectedness of motor and ocular motility. Hence, including evaluation of visual and motor proficiencies at school age would help to detect struggles in these crucial areas of development.

## Introduction

Visual and motor deficits can present in several developmental disorders such as developmental coordination disorder (DCD), attention-deficit/ hyperactivity disorder (ADHD), learning disabilities, dyslexia, autism spectrum disorder (ASD) or sensory processing disorder, among others (2; 8; 28; 40, 41; 49) and interfere with the performance of daily activities. Therefore, increasing knowledge about the interrelationship of both development areas could facilitate early detection and the implementation of appropriate interventions.

Motor skills are fundamental for a child's overall development and can influence other seemingly unrelated areas. For instance, there is evidence of a profound interconnection between motor and cognitive skills (10) at the level of underlying brain structures, where one can influence the other (36). Additionally, several studies have highlighted the impact of motor proficiency on academic achievements (1; 25), and the association between fine motor skills and social competence (11). Moreover, contrary to what is believed, these motor difficulties may persist as children grow (24; 8). Therefore, they may be more likely to experience challenges in other development domains, which justifies early assessment.

The prevalence of DCD, according to the Diagnostics and Statistical Manual of Mental Disorders (DSM-5), is around 5-6% in children between 5 and 11 years old (4). However, recent studies carried out in Spain estimate that between 12.2% and 17.4% of school children can present suspected DCD or are at risk and suggest that DCD is an underdiagnosed disorder in Spain (3; 12). Besides, the DCD can co-occur with ADHD, specific language impairment (especially reading and written expression) and ASD (4).

Moreover, the visual system's development is intricately intertwined with motor development during childhood. Both systems improve as children grow older, and integrating these two developmental areas is essential for activities that require hand-eye coordination, spatial awareness, and overall cognitive development. Several authors have examined the relationship between binocular vision and motor performance, indicating that conditions like strabismus and amblyopia are associated with impaired motor development (6; 26). Additionally, recent research points out that the visual system has a complex influence on postural control, as different eye movements elicit varying postural responses (30).

In recent years, various fields of knowledge have used eye tracking technology to record and analyse eye movements, positions, and gaze points. This method enables valuable data collection about performance in natural or laboratory settings (13; 15; 18; 31; 37; 42; 43; 47; 50). Furthermore, the use of eye-tracking devices in ophthalmology and optometry has grown exponentially in recent times and has been employed in various areas such as the evaluation, treatment, and analysis of diverse ocular disorders, including strabismus, amblyopia, nystagmus and the sequelae of concussion (19; 33).

In addition, the relationship between eye movements and reading is well documented in the literature. Recent studies using the eye tracker show that poor readers present abnormal patterns in eye movements compared to children with average or above reading ability (21; 48; 47). Furthermore, individuals with poor reading skills, diagnosed with Dyslexia or not, experience more significant motor coordination challenges than proficient readers (23). These data highlight the necessity of interdisciplinary assessments for a comprehensive approach to development.

Finally, recent studies show that visual skills such as accommodation and binocular vision impact motor performance (26; 27; 41). Therefore, research could benefit from exploring the relationships between motor skills and a broader range of visual abilities, including ocular motility (saccades, fixations, and pursuits).

The present study aims to improve understanding of the relationship between eye movements and motor skills. We hypothesised that children with better oculomotor abilities would present higher motor performance and vice versa. We examine how eye movement variables are linked to motor test scores in first and second-grade children.

## Methods

### Participants

This descriptive cross-sectional study was conducted in two schools located in Zaragoza, Spain. Prior to their participation, the parents or legal guardians of the students had to provide informed consent. The research received ethical approval from the Ethics Committee of Aragon (CEICA) under reference (PI22/459) and it adheres to the principles outlined in the Declaration of Helsinki.

The inclusion criteria were as follows: first and second-grade students whose guardians provided informed consent. Additionally, participants were required to understand and perform the specified tests in the study. The following individuals were excluded: those who showed cooperation difficulties and those who could not complete all the tests for other reasons, such as severe visual impairment.

This study's data come from a sample of 174 children, but only 142 (62 girls and 80 boys) met the inclusion criteria and completed all visual and motor tests. This avoided missing data for all variables. Their mean age was 7.08 ± 0.61 years.

### Materials

The Developmental Eye Movement (DEM) and Northeastern State University College of Optometry's Oculomotor test (NSUCO) tests are commonly applied in optometric evaluation of ocular motility (21; 32; 34). In this study, eye tracking was employed to obtain objective measurements of various eye movement parameters (saccades, regressions, fixations, as well duration of fixations) during the DEM test.

DEM is a visual-verbal assessment test designed to indirectly evaluate saccadic eye movements. It begins with a preliminary assessment phase to determine the child's familiarity with numbers and its ability to perform the test. The test comprises two cards (Test A and B) with 40 numbers presented in two columns, which the subject must read vertically. Additionally, there is a card containing 80 numbers arranged horizontally in 16 rows with variable spacing (Test C). The subject is asked to read the numbers as quickly as possible while minimising errors. The time taken for each card and the mistakes made (addition, omission, substitution, transposition) are recorded. The horizontal time should be adjusted based on the number and type of errors, since the difference between reading 80 numbers arranged vertically and the same set of digits arranged horizontally is evaluated. The ratio is computed by dividing the adjusted horizontal time by the vertical time. Subsequently, values for horizontal and vertical times, ratio, and the number of errors are converted into percentiles (14).

Eye tracking was executed using the Tobii Eye X eye tracker (Tobii, Stockholm, Sweden) during the DEM test, and data analysis was performed through Clinical Eye Tracker software (Thomson Software Solutions, Welham Green, UK). The eye tracker is a device that objectively records eye position and movement during different visual tasks by detecting the pupil and corneal reflex using infrared light. The eye tracking device was positioned approximately 60 cm from the subjects, adjacent to a 23.8-inch auxiliary screen. The eye tracker boasts a sampling rate of 55 Hz (17). The eye tracker was calibrated for each participant before administering the DEM test.

The NSUCO test is a standardised evaluation that subjectively assesses tracking and saccadic eye movements. The assessment is conducted by observing how the patient, who stands facing the examiner, performs eye movements during the test. In the pursuits test, a spherical fixation point, approximately 0.5 cm in size, is placed at a distance of about 40 cm. While the subject maintains fixation on this object, two circles with a diameter of about 20 cm are traced clockwise, followed by two counterclockwise rotations. For the saccadic eye movement test, two fixation points spaced approximately 20 cm apart are used. The subject, standing about 40 cm away, is required to alternate their gaze between these points, completing ten cycles. Scores ranging from 1 to 5 points are assigned to evaluate the subject's ability, precision, and head and body movements during the test, depending on their performance. This test, therefore, evaluates how well the eyes can move independently from the head and body movements. (32).

The Spanish version of the Movement Assessment Battery for Children-2 (MABC-2) was used to determine the children’s degree of motor performance (20). The test is administered according to three age bands (age band 1: 4–6 years, age band 2: 7–10 years, age band 3: 11–16 years), each with eight age-appropriate test items grouped into three motor domains: manual dexterity, aiming and catching, and balance. The raw score for each item is converted into a scaled score with a mean of 10 and a standard deviation of 3. The score for each domain is obtained by adding the corresponding items and is converted again into a scaled score (M=10; SD=3) and percentiles. The sum of the three domains provides the test's total score, which is again transformed into a scaled score and percentile. The 5th and lower percentiles indicate that the child has severe movement difficulties, and scores between the 6th and 15th percentiles represent children with borderline motor impairment. The often-standard cut-off value of 15% is unavailable in the MABC-2 (46). However, the authors provide the correspondence between the 5th and 15th percentiles and the total test score, 59 and 68 points, respectively. Only items designed for age bands 1 and 2 were used in this study.

### Procedure

After consulting with principals, the investigators explained the study's objectives and provided information in a meeting with teachers and families. Adequate space for evaluations was provided by each school.

Visual function assessments were conducted by three optometrists, encompassing the following tests: visual acuity for both far and near vision, objective refraction utilising retinoscopy, cover tests for far and near vision, near point of convergence, Worth's test, stereopsis evaluation using the Random Dot test, NSUCO test, horizontal vergences measured at steps for both far and near vision, vergence facility employing ±2.00 D lenses, accommodative amplitude determined via the Donders method, and monocular as well as binocular accommodative facility. A digitised version of the DEM test was also created to gather the participants' performance through an eye tracker.

Concurrently, five occupational therapists conducted motor performance assessments using the MABC-2 test. It was administered individually and took between 20 and 30 minutes.

### Data Analysis

The measurements were recorded in a database created with Microsoft Office Excel 2016. Statistical analysis for each variable was conducted using a tool based on the R programming language, specifically designed for statistical applications.

For statistical analysis, subjects were categorised into three groups (Group 1: Significant movement difficulty, Group 2: “At risk” of developing motor difficulties, Group 3: Typical Motor Development) based on their total test score (<60, 60-68, >68) obtained from the MABC-2.

The Kolmogorov-Smirnov test was used to compare the distribution of variables to assess normality. Since most of the variables did not follow a normal distribution the non-parametric Wilcoxon-Mann-Whitney test was employed to compare the distribution of various variables among the three independent groups, with a significance level set at 0.05. The degree of association between quantitative variables was determined through Spearman's correlation with Holm-Bonferroni correction, characterised by the correlation coefficient provided by the aforementioned test. All these statistical tests were performed at a significance level of 95%. The minimum power for the different tests used with our sample size was established at 0.8.

**Figure 1.**
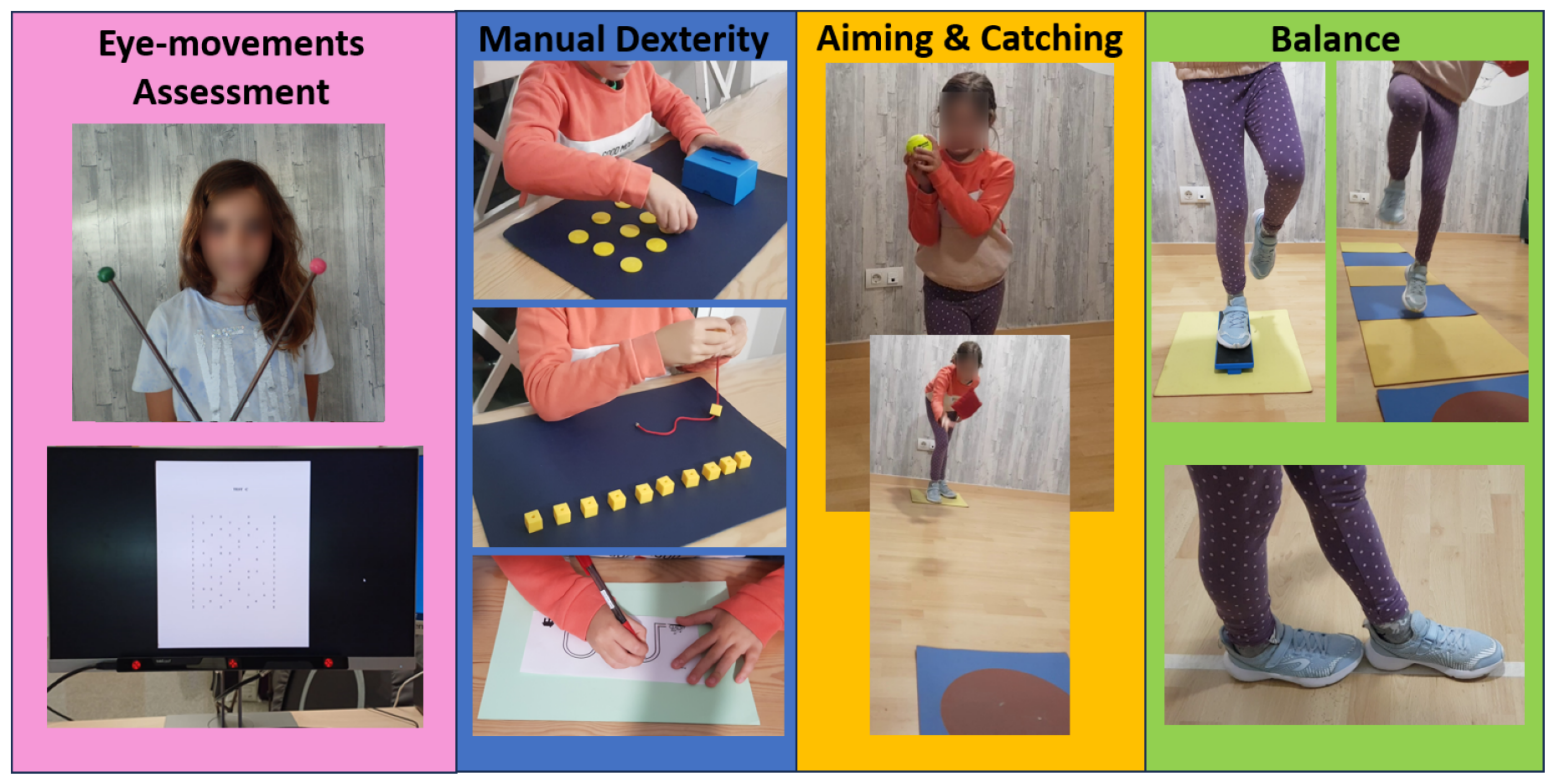
Examples of tasks to assess eye movements and motor performance.

## Results

Percentiles for vertical time, adjusted horizontal time, and the number of errors were obtained using the software included in the DEM test. Furthermore, Table 1 presents the data concerning parameters obtained through the eye tracker during the horizontal Test C. Additionally, the findings from the Wilcoxon test are displayed. Statistically significant differences have been emphasised in bold typeface.

**Table 1.** Results of the DEM test of the total sample and the groups selected according to motor performance.

	Total M±IQR	G1 M±IQR	G2 M±IQR	G3 M±IQR	*p value*		
**DEM**	N=142	n=28	n=21	n=93	**G1vsG2**	**G2vsG3**	**G1vsG3**
**Vertical Time (pctl)**	28±51.5	5±26.5	17±26.549	36±45.5	0.061	0.098	**<0.001**
**Horizontal Time (pctl)**	37.5±51.5	11.5±29	26±43	45±44	0.145	0.165	**<0.001**
**Errors (pctl)**	28±50	19.5±36.75	28±36.75	36.5±48.75	0.650	0.498	0.142
**Duration test C (s)**	86.4±38.625	99.85±43.225	94.3±43.9	81.7±35.775	0.332	0.116	**0.005**
**Number of saccades (n)**	107±46.75	111.5±50.5	106±57	106±46.25	0.524	0.734	0.224
**Regressions (n)**	56.5±35.5	70±44.25	53±42	53±30.5	0.160	0.348	**0.003**
**Fixations (n)**	164±80.75	176.5±68.25	148±95	159±69	0.266	0.555	**0.034**
**Duration fixations (ms)**	443±138	445±136	437±191	443±138	0.709	0.585	0.701

Note. G1: Group 1 (Significant movement difficulty), G2: Group 2 (“At risk” of developing motor difficulties, G3: Group 3 (Typical motor development). M: Median, IQR: Interquartile Range, pctl: percentile, s: seconds, ms: milliseconds.

The relationship between total scores on the MABC-2 and performance on the DEM test is evident. A higher total score on the MABC-2 corresponds to better performance on the DEM test. In the group with typical motor development, we observed higher percentiles for both vertical and horizontal time, fewer errors, and faster test performance. Additionally, this group's average number of saccades, fixations and regressions decreased. Concerning fixation duration, it was generally lower in the group with the poorest motor performance. Statistically significant differences were found between most parameters of Group 1 and Group 3, while no significant differences were observed between Group 2 and the other groups.

Table 2 presents the values of the NSUCO test. The results of the Wilcoxon test are displayed, with statistically significant differences highlighted in bold typeface.

For most parameters, the group with typical motor development scored closer to 5, which represents the highest score in the NSUCO test compared to the other groups. Statistically significant differences were observed between most parameters of Group 1 and Group 3, as well as some differences between Group 2 (“at risk”) and Group 3.

**Table 2.** Results of the NSUCO test of the total sample and the groups selected according to motor performance.

		Total M±IQR	G1 M±IQR	G2 M±IQR	G3 M±IQR	*p value*		
	**NSUCO**	N=142	n=28	n=21	n=93	**G1vsG2**	**G2vsG3**	**G1vsG3**
PURSUITS	**Ability**	5±0	5±1	5±0	5±0	0.761	0.399	0.098
	**Accuracy**	4±2	3±2.25	4±2	4±2	0.3349	0.177	**0.003**
	**Head Movement**	24±2	4±2.25	4±3	4±1	0.917	**0.046**	**0.033**
	**Body Movement**	5±1	5±1	5±1	5±0	4±1	0.101	**0.036**
SACCADES	**Ability**	5±1	5±1	5±1	5±0	0.549	0.110	0.249
	**Accuracy**	4±3	3±2.25	3±2	4±2	0.483	**0.017**	**<0.001**
	**Head Movement**	4±2	3±3	2±3	4±3	0.884	**0.013**	**0.017**
	**Body Movement**	5±1	4±1	5±1	5±1	0.369	0.541	0.075

Note. G1: Group 1 (Significant movement difficulty), G2: Group 2 (“At risk” of developing motor difficulties, G3: Group 3 (Typical motor development). M: Median, IQR: Interquartile Range,

Table 3 highlights values with p <0.05 in bold, indicating correlations between various ocular motility parameters obtained from the DEM test and data from the MABC-2 test. The table also displays the correlation coefficients between different variables.

**Table 3.** Correlations between MABC-2 test and DEM test.

		**Vertical Time (pctl)**	**Adjusted Horizontal Time (pctl)**	**Errors (pctl)**	**Duration Test C**	**Number saccades**	**Number Regression**	**Number fixations**	**Duration fixations**
**Total Score**	CC	0.338	0.314	0.076	-0.263	-0.170	-0.282	-0.237	-0.006
	*p*	**0.000**	**0.000**	0.366	**0.002**	**0.043**	**0.001**	**0.005**	0.943
**Manual Dexterity**	CC	0.226	0.179	0.029	-0.171	-0.166	-0.231	-0.216	0.082
	*p*	**0.007**	**0.034**	0.732	**0.042**	**0.049**	**0,.06**	**0.010**	0.330
**Aiming & Catching**	CC	0.184	0.203	0.046	-0.188	-0.031	-0.117	-0.073	-0.103
	*p*	**0.029**	**0.015**	0.590	**0.025**	0.712	0.165	0.388	0.223
**Balance**	CC	0.320	0.268	0.078	-0.205	-0.142	-0.251	-0.201	0.020
	*p*	**0.000**	**0.001**	0.359	**0.014**	0.092	**0.003**	**0.016**	0.814

Note. CC: correlation coefficient. Pctl= percentile.

While correlations exist between several parameters, they tend to be weak or moderate. Positive correlations were found between most motor tests and vertical and horizontal time percentiles, indicating that better motor performance corresponds to better DEM test performance. Negative correlations were observed between motor tests and the number of saccades, regressions, and fixations, suggesting that a higher number of these eye movements may indicate less efficient ocular motility. However, no correlations were found between motor test values and the number of errors or fixation duration.

Table 4 illustrates correlations between MABC-2 and certain NSUCO test parameters, although the correlation coefficients are generally low. All correlations identified are positive, suggesting that better motor performance is associated with greater precision in both saccades and pursuits. Furthermore, a greater dissociation between eye movements and head or body movements corresponds to better motor performance.

**Table 4.** Correlations between MABC-2 test and NSUCO test.

		**Pursuits**				**Saccades**			
		**Ability**	**Accuracy**	**Head Movement**	**Body Movement**	**Abilit**	**Accuracy**	**Head Movement**	**Body Movement**
**Total Score**	CC	0.133	0.243	0.203	0.224	0.158	0.372	0.269	0.158
	*p*	0.114	**0.004**	**0.015**	**0.007**	0.061	**0.000**	**0.001**	0.060
**Manual Dexterity**	CC	0.112	0.110	0.179	0.196	0.093	0.298	0.244	0.132
	*p*	0.184	0.192	**0.033**	**0.019**	0.270	**0.000**	**0.003**	0.117
**Aiming & Catching**	CC	0.039	0.198	0.066	0.117	0.079	0.186	0.097	0.083
	*p*	0.645	**0.018**	0.433	0.165	0.347	**0.027**	0.253	0.326
**Balance**	CC	0.154	0.206	0.159	0.183	0.144	0.280	0.250	0.161
	*p*	0.067	**0.014**	0.059	**0.029**	0.088	**0.001**	**0.003**	0.055

Note. CC: correlation coefficient.

## Discussion

The primary aim of this study was to examine the relationship between ocular motility and motor skills in school-age children. We assessed eye movement with the NSUCO test and recorded eye movements during the DEM test through an eye tracker. Additionally, these first and second-grade children were classified according to their motor performance in the MABC-2. Findings increase knowledge of the complex interplay between visual and motor domains and may be helpful for professionals from different fields.

Hand-eye coordination and ocular motility are pivotal for catching, grasping, object manipulation, and aiming and targeting tasks. Pursuits allow tracking of moving objects and saccades shift focus from one fixation point to another. Both eye movements facilitate the precise alignment of the fovea with the object of interest. Eye movements precede motor actions because vision provides essential information about our surroundings, including the location, size, and characteristics of objects, enabling us to plan movements, such as reaching, grasping, walking, and going upstairs (29).

Our findings suggest that ocular motility plays a crucial role in hand-eye coordination. Various parameters from the DEM test, which indirectly assesses small-amplitude saccades and the NSUCO test, which evaluates pursuits and large-amplitude saccades, demonstrate correlations with the outcomes of the MABC-2. While these correlations exhibit weak or moderate correlation coefficients, they indicate that individuals with superior ocular motility tend to perform better in specific motor skills.

Tasks involving aiming and catching demand spatial mastery. When a child is asked to aim at a target, movement planning is required to locate the target in space and accurately throw the object, whether a ball or a beanbag. During catching tasks, the visual and motor systems interact before the motor action. Therefore, visual object tracking is essential to anticipate the movement and appropriately position both the body and upper limbs to intercept the moving object (24).

Groups 1 and 2 presented significant differences compared to group 3 (typical motor development) regarding head movements in the NSUCO test. Eyes and head movements must be coordinated to meet the demands of different tasks and environments. Therefore, they must be flexible and can be coupled or uncoupled. Research indicates that even young children stabilise their heads before reaching (44) and that poor readers tend to make a more significant number of movements and a greater head span than good readers (32); so during reading head movements are suppressed to allow stable fixation (39). Therefore, children in the “risk group” may struggle to perform adequately in school.

Our study observed that a higher number of saccades, regressions, and fixations, along with poorer scores on ocular motility tests, were associated with lower motor performance. Few studies have explored the relationship between ocular motility, specifically saccades and pursuits, and motor performance in children. Some previous investigations have focused on patients with strabismus and amblyopia. Kelly et al. (27) conducted a study examining saccades and temporal hand-eye coordination during a simple reaching task on a touch screen in 7- to 12-year-old children with esotropia, who had previously been treated for strabismus. They discovered that children with strabismus exhibited altered saccadic kinematic and slower reaching during the final stages of oculomotor coordination. Consequently, these children might struggle to adopt an efficient strategy during visually guided reaching tasks. Saccade latency was longer in strabismic children compared to controls, and the accuracy of primary saccades was approximately 25% lower, with final saccades exhibiting a 45% lower accuracy. Furthermore, the absence of stereopsis was associated with reduced precision in primary saccades and a higher number of saccades related to reaching. This study suggests that binocular dysfunction leads to slower reaching movements. In addition, children with strabismus show a less accurate and longer deceleration phase. Stereoacuity also plays a role in precisely locating objects in space, influencing the deceleration phase of hand movement when attempting to touch a target on the screen.

It is essential to note that while some authors have supported the DEM test for assessing ocular motility, especially saccadic movements (16; 51), others have raised concerns due to the involvement of verbal and visual processing skills (5; 35). Since the standard parameters provided by the test (vertical time, adjusted horizontal time, ratio, and number of errors) do not appear to be directly linked to ocular motility (5; 38). However, several studies have identified a correlation between reading ability and DEM test results.

In our study, the vertical and horizontal DEM percentiles and test C duration correlate with the total motor score and the three dimensions from the MABC-2. Hopkins et al. (22), who evaluated 222 Grade 2 children, showed that Visual-motor integration (VMI) and the vertical and horizontal DEM times were associated with reading and mathematics performance. Besides, recent Eye-tracking studies have shown that shorter fixations during reading correlate with higher reading speed (48; 21). Furthermore, in the study by Hindmarsh et al. (21), second-grade children completed a standardised reading comprehension test and a computerised version of the DEM test while their eye movements were recorded. They observed that the characteristic which most differentiated the eye movement behaviour between children exhibiting below-average and average or above-average reading ability was the proportion of inter-line eye movements (vertical movements that shifted fixation away from the current line). These movements were more frequent in the group with lower reading abilities. On the other hand, Iversen's (23) study showed that children aged 10 to 12 with dyslexia and poor reading abilities performed worse than good readers on manual dexterity and balance tasks on the MABC. Hence, a multidisciplinary and multifaceted evaluation from a broad, dynamic perspective is indispensable. This approach is essential due to the intricate relationship between different areas of development.

Other visual skills are also related to motor performance. Binocularity is crucial for tasks like judging distances and perceiving three-dimensional shapes, significantly impacting hand-eye coordination's accuracy, speed, and efficiency. Poor binocular vision, as seen in conditions like amblyopia and strabismus, correlates with impaired motor performance due to compromised stereopsis, essential for spatial perception. (6; 26). Rafique and Northway (40) assessed motor skills and accommodative control in children with DCD, and their findings revealed a correlation between accommodative dysfunctions and the performance of visuomotor tasks involving upper extremities and fine dexterity.

Finally, our results emphasise the association between saccadic and pursuit eye movements and motor performance. Coetzee and Pienaar (9) assessed the impact of an 18-week visual training program on a group of children with DCD. This program significantly improved visual skills related to visual search, fixation, and convergence, ultimately leading to enhanced scores in the MABC-2. Similarly, other researchers have observed that improving eye movements can enhance performance in activities such as object throwing and catching and overall motor coordination (45; 52). Furthermore, eye movements contribute in a complex way to postural control since performing saccadic movements during balance tasks causes a decrease in sway, unlike visual pursuit (30). Thus, understanding how different systems influence each other can facilitate the implementation of appropriate interdisciplinary interventions.

This study is not without limitations. The size of the groups with poorer and borderline motor performance is small. The results of this study should be corroborated with a larger sample in the groups with worse motor performance. Participants attended two schools in the same neighbourhood, and we used non-probability convenience sampling, which may have implied a selection bias and limited the results' generalizability. However, a state-funded school and another state-subsidized private school from different socioeconomic levels were selected to cover a more diverse population. Finally, this study did not consider sociodemographic and contextual variables. Future studies should explore the associations between those confounding variables and motor-visual development.

Our results support previous research findings by emphasising the connection between motor and visual skills. Therefore, it is essential to assess these skills thoroughly during school years. Early identification of motor and visual struggles can help prevent learning difficulties and support the child's overall development.

### Ethics and Conflict of Interest

The author(s) declare(s) that the contents of the article are in agreement with the ethics described in http://biblio.unibe.ch/portale/elibrary/BOP/jemr/ethics.html and that there is no conflict of interest regarding the publication of this paper.

### Acknowledgements

The authors thank all the children and families for participating in this study and the teachers and principals for their help and collaboration.
